# Changes in circulating extracellular vesicle cargo are associated with cognitive decline after major surgery: an observational case–control study

**DOI:** 10.1016/j.bja.2024.07.040

**Published:** 2024-10-18

**Authors:** Souren Mkrtchian, Maria Eldh, Anette Ebberyd, Susanne Gabrielsson, Ákos Végvári, Sven-Erik Ricksten, Mattias Danielson, Jonatan Oras, Andreas Wiklund, Lars I. Eriksson, Marta Gómez-Galán

**Affiliations:** 1 Department of Physiology and Pharmacology, Section for Anaesthesiology and Intensive Care Medicine, Karolinska Institutet, Stockholm, Sweden; 2 Division of Immunology and Respiratory Medicine, Department of Medicine Solna, Karolinska Institutet, Stockholm, Sweden; 3 Center for Molecular Medicine, Karolinska University Hospital, Stockholm, Sweden; 4 Clinical Immunology and Transfusion Medicine, Karolinska University Hospital, Stockholm, Sweden; 5 Division of Chemistry I, Department of Medicinal Biochemistry and Biophysics, Karolinska Institutet, Stockholm, Sweden; 6 Department of Anesthesia and Intensive Care, Sаhlgrenska University Hospital, Gothenburg, Sweden; 7 Department of Anesthesiology and Intensive Care Medicine, Institute of Clinical Sciences, Sahlgrenska Academy, University of Gothenburg, Sweden; 8 Capio Artro Clinic, Stockholm, Sweden; 9 Function Perioperative Medicine and Intensive Care, Karolinska University Hospital, Stockholm, Sweden

**Keywords:** circulating extracellular vesicle, miRNA, orthopaedic surgery, postoperative cognitive decline, proteomics

## Abstract

**Background:**

Postoperative neurocognitive decline is a frequent complication triggered by unclear signalling mechanisms. This observational case–control study investigated the effects of hip or knee replacement surgery on the composition of circulating extracellular vesicles (EVs), potential periphery-to-brain messengers, and their association with neurocognitive outcomes.

**Methods:**

We mapped the microRNAome and proteome of plasma-derived EVs from 12 patients (six with good and six with poor neurocognitive outcomes at 3 months after surgery) at preoperative and postoperative timepoints (4, 8, 24, and 48 h). Complement C3–EV association was confirmed by flow cytometry in plasma- and cerebrospinal fluid (CSF)-derived EVs, with total plasma and CSF C3 and C3a concentrations determined using enzyme-linked immunosorbent assay.

**Results:**

Differential expression analysis found eight dysregulated EV microRNAs (miRNAs) exclusively in the poor neurocognitive outcomes group. Pathway analysis suggested potential downregulation of proliferative pathways and activation of extracellular matrix and inflammatory response pathways in EV target tissues. Proteome analysis revealed a time-dependent increase in immune-related EV proteins, including complement system proteins, notably EV surface-associated C3. Such upward kinetics was detected earlier in the poor neurocognitive outcomes group. Interestingly, CSF-derived EVs from the same group showed a drastic drop of C3 at 48 h with unchanged concentrations in the good neurocognitive outcomes group. Functionally, the complement system was activated in both patient groups in plasma, but only in the poor neurocognitive outcomes group in CSF.

**Conclusions:**

Our findings highlight the impact of surgery on plasma- and CSF-derived EVs, particularly in patients with poor neurocognitive outcomes, indicating a potential role for EVs. The small sample size necessitates verification with a larger patient cohort.

**Editor's key points**

• Postoperative neurocognitive decline is triggered by unclear signalling mechanisms.
• The authors investigated the effects of surgery on circulating extracellular vesicles, potential mediators of cognitive dysfunction, and their association with neurocognitive outcomes.
• Differential expression analysis showed eight dysregulated microRNAs exclusively in the poor neurocognitive outcomes group, potentially linked to the downregulation of proliferative pathways and the activation of inflammatory response pathways.
• Proteome analysis showed increases in immune-related proteins, including members of the complement system.
• These findings suggest that surgical trauma reprograms expression of plasma extracellular vesicle-associated microRNAs and proteins in patients experiencing postoperative neurocognitive decline.

Higher brain functions related to neurocognitive processes are often impaired by surgery and trauma. Long-term (>3 months) postoperative neurocognitive disorder with a risk for later permanent dementia is now globally recognised as a major health concern.^1^^,^^2^ However, the pathophysiological mechanisms and signalling pathways involved remain poorly understood.

Rapid activation of the local and systemic innate immune system with the ensuing transmission of inflammatory humoural and cellular mediators to the brain has been suggested as an early triggering mechanism behind postoperative neurocognitive functions.^3^ This was recently confirmed in studies showing a pronounced increase in systemic and cerebrospinal fluid (CSF) biomarkers for inflammation and neurodegeneration.^4^^,^^5^ Moreover, surgery-induced neuroinflammation correlates strongly with long-term (3-month) postoperative neurocognitive outcomes.^4^ While inflammation-driven signalling remains a plausible hypothesis for early perturbations related to cognitive function, other periphery-to-brain signalling mechanisms with a potential for more long-term impact need to be considered.

A novel emerging class of intercellular messengers known as extracellular vesicles (EVs) is hypothesised to convey signals from the site of release to target tissues including the brain.^6^, ^7^, ^8^ The regulatory function of EVs is accomplished by various EV cargo molecules including microRNAs (miRNA), proteins, messenger RNAs (mRNAs), and others with the potential to modify intracellular metabolism and signalling upon the uptake of EVs by target cells.^8^ This led to our hypothesis that surgery-triggered release of EVs actively contributes to mechanisms related to long-term neurocognitive disorder. Supported by our recent preclinical data confirming that orthopaedic surgery influences expression of cargo molecules (miRNA and proteins) in mouse circulating EVs,^9^ our primary aim was to map postoperative microRNAome and proteome profiles of circulating EVs in human plasma samples. The secondary aim was to explore the association between changes observed at the miRNA and protein concentrations with postoperative neurocognitive outcomes reported at 3 months after the surgery.

## Methods

### Patients and study design

This study is a follow-up to a longitudinal prospective observational, case–control study (NEUPORT)^4^^,^^5^ and includes 12 out of a total of 27 patients subjected to elective knee/hip replacement under spinal anaesthesia as approved by the Regional Ethics Committee in Stockholm, Sweden (Dnr 2013/2297–31/4, 2014/834–32, and 2022-06783-02; NCT02759965). Patient selection was based on rigorous inclusion criteria including a preoperative Mini-Mental State Examination (MMSE; patients with <24 MMSE scores were excluded).^4^

### Neurocognitive testing and group selection

Preoperative (1–2 weeks before surgery) and postoperative (3–5 days and 3 months after surgery) neurocognitive capacities in all 27 patients were evaluated using the International Study of Postoperative Cognitive Dysfunction (ISPOCD) test battery including seven test variables.^10^ We compared changes in performance for each test from baseline (preoperative) to 3–5 days and 3 months after surgery by quantifying a *z*-score for each variable and combining them into a composite *z*-score.^4^ Patients with a composite *z*-score of ≥1 at 3 months after surgery were assigned to the poor neurocognitive outcome group, whereas those with a composite *z*-score <1 were included in the good neurocognitive outcome group.^4^^,^^10^ At 3 months after surgery, only 6 out of the 27 patients showed a composite *z*-score >1. These six patients constituting the poor outcomes group (study subjects) and the six patients from the good outcomes group with the best (lowest) composite *z*-scores (control subjects) were chosen for the current study. Demographics, comorbidity, preoperative MMSE scores, patient perioperative characteristics, and averaged composite *z*-scores for each group are presented in Table 1. Individual composite *z*-scores representing changes in cognitive capacity from the postoperative to 3–5 days and 3-month concentrations are presented in Supplementary Figure 1. Additional details for all 27 patients' characteristics and inclusion and exclusion criteria can be found in two previous publications.^4^^,^^5^

**Table 1:** Patient characteristics and postoperative cognitive score by neurocognitive outcome at 3 months. Values are median (Q1–Q3) or *n* (%) or *n*. Group differences were tested by Mann–Whitney *U*-test or two-tailed unpaired t-test (∗∗∗∗*P*<0.0001). A two-way analysis of variance for repeated measures was used to assess differences between groups for severity of postoperative pain (VAS). ASA, American Society of Anesthesiologists; MMSE, Mini-Mental State Examination; PACU, post-anaesthesia care unit; VAS, Visual Analogue Scale; WBC, white blood cell. MMSE and composite *z*-score (see the ‘Methods’ section). MMSE and composite z-score were calculated as in refs.^4^, ^10^, respectively.

Variables	Good neurocognitive outcome (*n*=6)	Poor neurocognitive outcome (*n*=6)	*P*-value
**General**			
Age (yr)	71 (65–78)	68 (65–71)	0.16
Sex, woman	3 (50)	5 (83)	0.26
Weight (kg)	84 (63–101)	80 (65–93)	0.63
Body mass index (kg m^−2^)	26.0 (22.6–30.1)	28.0 (26.3–32.3)	0.21
**Comorbidities**			
Hypertension	4	4	>0.99
Diabetes mellitus, type 2	0	1	0.34
Nicotine use	0	0	>0.99
**ASA physical status**			
1/2/3/4	1/5/0/0	0/6/0/0	0.34
**Preoperative laboratory results**			
Blood haemoglobin (g L^−1^)	137 (112–156)	145 (130–168)	0.36
Serum creatinine (μM)	82 (66–97)	71 (40–105)	0.37
Preoperative WBC count (<10^9^ L^−1^)	6.1 (5.7–8.0)	7.5 (5.0–8.8)	0.1
**Intraoperative data**			
Spinal anaesthesia	6 (100)	6 (100)	
Propofol (mg)	285 (30–400)	176 (0–358)	0.21
Fentanyl	1	0	0.36
Alfentanil	2	0	0.15
Vasopressor use	2	5	0.09
Intravenous fluids (ml)	950 (400–1300)	1133 (800–2200)	0.51
Blood transfusion	0	1	0.34
Duration of procedure (min)	82 (65–100)	88 (70–112)	0.5
**Procedure**			
Hip replacement	4	5	0.55
Knee replacement	2	1	0.55
Blood loss (ml)	171 (0–350)	287 (100–600)	0.25
**Postoperative, 24 h**			
PACU length of stay (min)	200 (90–540)	377 (107–1360)	0.42
Intravenous fluids (ml)	2267 (900–3100)	2633 (950–4450)	0.54
**Medications**			
Gabapentin	1	2	0.55
Oral opioid	6	6	>0.99
Intravenous opioid	2	3	0.60
Post-spinal puncture	0	1	0.22
Blood patch	0	1	0.22
**Pain assessment, VAS score**			
4 h	3 (0–7)	1 (0–5)	0.21
8 h	4 (0–8)	5 (0–9)	0.71
24 h	6 (1–8)	6 (5–10)	0.54
48 h	5 (2–8)	6 (4–7)	0.53
**MMSE score**			
Preoperative	28.33 (25–30)	28.5 (27–30)	0.86
**Composite cognitive z-scores (change from preoperative to postoperative)**			
3–5 days	1.5 (–0.76 to –3.3)	2.9 (1.76–5.72)	0.1518
3 months	–1.3 (–0.54 to –2)	2 (1–3.7)	<0.0001∗∗∗∗

### Anaesthesia, blood, and CSF sample collection

**Fig 1:**
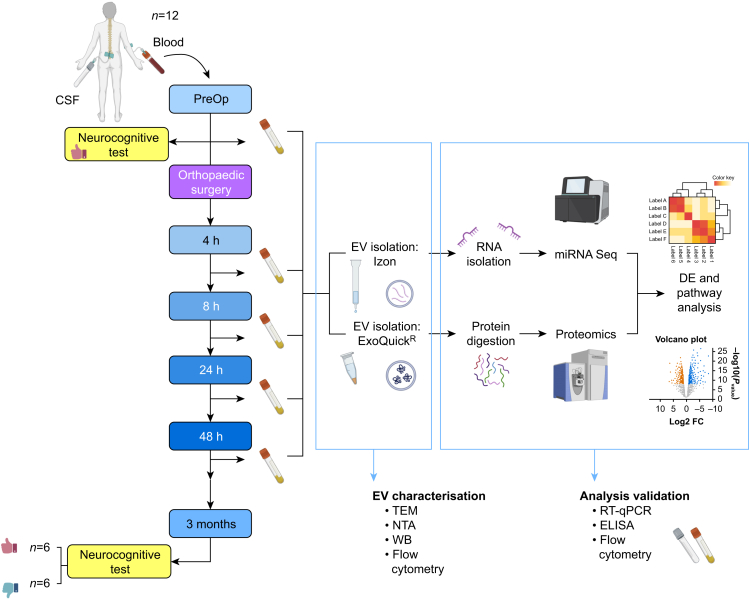
Study design and methodology. Hip or knee surgery was performed in patients under spinal anaesthesia supplemented by light sedation. Cerebrospinal fluid (CSF; 5 ml) and blood (20 ml) were collected preoperatively (PreOp) and at 4, 8, 24, and 48 h after skin incision. Preoperative (1–2 weeks before surgery) and postoperative (3 months after surgery) neurocognitive capacity was evaluated using the International Study of Postoperative Cognitive Dysfunction (ISPOCD) test battery (Supplementary Methods). Red thumbs up icon, patients with good neurocognitive function (*n*=6). Blue thumbs down icon, patients with poor neurocognitive function (*n*=6). The microRNAome and proteome of plasma-derived extracellular vesicles (EVs) isolated from 12 patients before and after the completion of orthopaedic surgery were mapped by RNA-sequencing and tandem mass spectrometry. EVs were characterised by standard methods and results were validated and analysed in both blood and CSF samples. DE, differentially expressed; ELISA, enzyme-linked immunosorbent assay; FC, fold change; miRNA, microRNA; TEM, transmission light microscopy; NTA, nanoparticle tracking analysis; WB, western blot; RT-qPCR, quantitative reverse transcription polymerase chain reaction.

After placement of an intrathecal catheter, the surgical procedure was performed under spinal anaesthesia supplemented by light sedation (Supplementary Methods). CSF and venous blood (20 ml) were collected preoperatively and at 4, 8, 24, and 48 h after skin incision. Serum and plasma were prepared as described,^4^ aliquoted, and stored at –80°C for subsequent analysis (Fig. 1).

### Extracellular vesicle isolation

Due to limiting plasma material, EVs were isolated from blood plasma using two approaches depending on the downstream analysis: (i) a commercial precipitation method for protein mass spectrometry and (ii) size-exclusion chromatography for RNA-seq.

Precipitation: Isolation of EVs was performed with ExoQuick ULTRA (System Biosciences Inc., Mountain View, CA, USA) according to the manufacturer's protocol as described^9^ (also Supplementary Methods).

Size-exclusion chromatography: We used 70-nm qEVoriginal size exclusion columns (Izon 70; Izon Science Limited, Christchurch, New Zealand) according to the manufacturer's protocol (Supplementary Methods).

EVs from CSF were isolated using the immunoaffinity approach. Briefly, aldehyde/sulfate latex beads (Invitrogen, Thermo Fisher Scientific, MA, USA) were incubated with 250 μl of CSF, and beads–EV complexes were then analysed for the presence of CD81 and C3 by flow cytometry (Supplementary Methods).

### Nanoparticle tracking analysis (NTA)

The particle concentration and size distribution of plasma-derived EVs were measured using the Nanosight LM10HSB system (NanoSight, Amesbury, UK) (Supplementary Methods).

### Negative stain transmission electron microscopy (nsTEM)

Electron microscopy analysis was conducted at the electron microscopy unit EMil (Karolinska Institute) as described^9^ (Supplementary Methods).

### Small RNA sequencing

Total RNA was extracted from iZon70-isolated EVs (Izon-EVs) using the Exosomal RNA isolation kit from Norgen Biotek Corp (Nordic BioSite AB, Täby, Sweden).

Library construction, quality control, and sequencing were conducted at the Genomics Core Facility of the Institute of Molecular Biology GmbH (IMB), Mainz, Germany (Supplementary Methods). Data processing was performed by in-house scripts (Supplementary Methods). The resulting matrix of raw read counts was used for downstream data analysis. Differential expression (DE) analysis was carried out using the online Bioconductor R package DEBrowser^11^ (Supplementary Methods). Principal component analysis (PCA), miRNA target analysis for each DE miRNA, and the corresponding pathway enrichment analysis are described in the Supplementary Methods.

### miRNA quantitative polymerase chain reaction

Validation of miRNA-seq results was accomplished by quantitative polymerase chain reaction (qPCR) of selected DE miRNAs using TaqMan Advanced miRNA Assays (Applied Biosystems, Thermo Fisher Scientific; Supplementary Methods).

### Proteomics

#### Liquid chromatography–tandem mass spectrometry–based proteome analysis

Quantification of relative protein abundances from ExoQuick ULTRA-isolated EVs (Exo-EVs) was performed using a tandem mass tag (TMT) technique by applying a multiplex approach. Sample preparation, peptide labelling with the TMT mass tag reagent, and subsequent separation of labelled peptides on EASY-Spray C_18_ column and mass spectra acquisition on Orbitrap Q Exactive HF mass spectrometer (Thermo Fisher Scientific) were performed at the Proteomics Biomedicum core facility (Karolinska Institutet [https://ki.se/en/mbb/proteomics-biomedicum]) as described in Supplementary Methods. Data normalization for DE analysis was conducted as described^12^ (Supplementary Methods). The normalized batch-corrected file was used for the analysis of differential protein abundance across timepoints per condition using the Bioconductor R package *limma* (Supplementary Methods).

#### Western immunoblot

Plasma-derived EVs and plasma depleted of EVs were solubilized with 2% sodium dodecyl sulfate. Samples containing equal amounts of protein were resolved by sodium dodecyl sulfate–polyacrylamide gel electrophoresis and transferred to a polyvinylidene difluoride membrane (Invitrogen, Thermo Fisher Scientific Supplementary Methods) for immunoblotting.

#### Enzyme-linked immunosorbent assay

Total complement C3 and C3a concentrations in the serum and CSF were determined using standard sandwich enzyme-linked immunosorbent assays (ELISAs; Supplementary Methods).

#### Plasma- and CSF-derived extracellular vesicle immunoaffinity capture and analysis by flow cytometry

Bead-based flow cytometry was performed as described.^13^ In brief, aldehyde/sulfate latex beads (Invitrogen, Thermo Fisher Scientific) were coated with anti-human CD9 antibody, incubated with Exo-EVs from plasma and whole CSF, and analysed for the presence of CD9, CD63, CD81, and C3 by flow cytometry (Supplementary Methods).

### Statistics

Statistical analyses (except for the -*omics* data analyses, which are described in the Supplementary Methods) were performed by two-way analysis of variance with repeated measures followed by uncorrected Fisher's least significant difference multiple comparisons test (GraphPad Prism version 10.1.0; GraphPad Software, Boston, MA, USA). Data are baseline-corrected by preoperative values per patient and log2-transformed before applying the statistical model. Data are presented as mean (sem). *P*<0.05 was considered statistically significant and *ns* refers to *P*<0.1.

## Results

The overall study design is presented in Figure 1. To discriminate between the global impact of surgery and the potential neurocognitive outcome-specific changes of miRNAome proteome in plasma-derived EVs, we divided the acquired data into three different groups: (i) combined, (ii) good, and (iii) poor (see the ‘Methods’ section).

### Extracellular vesicle isolation and characterization

EVs isolated by the two different methods (Izon70 [Izon-EVs] and ExoQuick ULTRA [Exo-EVs]) were characterised by nsTEM, NTA, flow cytometry, or western immunoblot (Supplementary Figs 2 and 3). No differences in particle size distribution between the different timepoints or between groups were detected between methods (Supplementary Figs 2c and 3c). However, there was a significant postoperative reduction in the concentration of normalised particles in both Izon- and Exo-EVs (Supplementary Figs 2d and 3d).

#### Extracellular vesicle miRNAome

**Fig 2:**
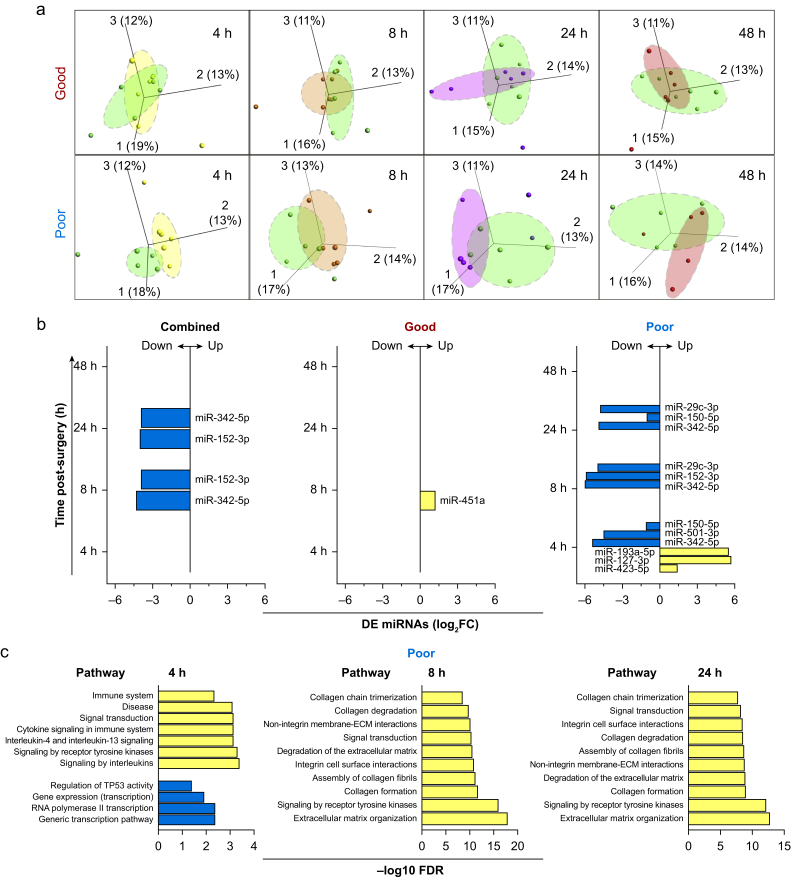
Differential expression (DE) of microRNAs (miRNAs) in circulating extracellular vesicles. (a) Principal component analysis (PCA) of circulating extracellular vesicles (EVs) miRNAome. CPM (counts per million)-normalised miRNA read counts were filtered for low-expression miRNAs, log2-transformed, and used as a matrix table by Qlucore software (QLUCORE, Lund, Sweden) to generate PCA plots. PCA plots for all timepoints in the good (upper panel) and poor outcome groups (lower panel). (b) DE of miRNAs in plasma-derived EVs from patients from the combined, good, and poor outcome groups. DE is estimated relative to preoperative values and miRNAs with a false discovery rate (FDR) ≤0.1 were considered statistically significant and referred to as DE miRNAs. (c) Pathway analysis of DE miRNA target messenger RNAs (mRNAs) in the poor outcome group. mRNAs for each of upregulated or downregulated miRNAs were searched in the Reactome database for enriched pathways. The top significantly (FDR ≤0.05) enriched pathways are shown. FC, fold change; Yellow, upregulated pathways; blue, downregulated pathways.

Next-generation RNA sequencing of Izon-EVs identified about 1400 miRNAs; after filtering out low-expressed miRNAs, this number was reduced to 280–290 miRNAs depending on the group (Supplementary Methods and Supplementary File A). PCA did not reveal time-dependent clustering in the combined group (data not shown). However, a per-group-based analysis revealed a pronounced separation at 4 h after surgery in the poor group (Fig. 2a). DE analysis of miRNA expression confirmed the PCA data (Fig. 2b). In the combined group, we found only two downregulated miRNAs (miR-342-5p and miR-152-3p) at 8 and 24 h after surgery. However, when analysing the two conditions separately we observed noticeable differences between the two groups. Thus, at 4 h after surgery, three miRNAs (miR-193a-5p, miR-127-3p, and miR-423-5p) were upregulated and three (miR-342-5p, miR-501-3p, and miR-150-5p) were downregulated exclusively in the poor outcome group. At 8 and 24 h, miR-342-5p remained strongly downregulated together with miR-152-3p (at 8 h), miR-150-3p (at 24 h), and miR-29c-3p (at 8 and 24 h). There were no DE miRNAs at 48 h after surgery. In contrast, only one DE miRNA was found upregulated (at 8 h after surgery) in the good group (miR-451a; Fig. 2b). The validity of sequencing data was verified by qPCR for some of the DE miRNAs from the combined and poor groups (Supplementary Fig. 4).

### Pathway analysis

To ascertain the potential regulatory functions of the DE miRNAs from the poor neurocognitive outcome group, we initially identified target mRNAs for each of the DE miRNAs (Supplementary Methods). After merging all target mRNAs identified separately for each DE miRNA at 4 h after surgery, we obtained a list of 79 mRNAs for the upregulated group and 46 for the downregulated one. Similarly, 105 and 110 mRNAs were identified for the groups of downregulated miRNAs at 8 and 24 h, respectively (Supplementary File B). Results presented in Figure 2c demonstrate pathways enriched by the predicted mRNA targets. Most of the enriched pathways associated with the mRNA targets of upregulated miRNAs (at 4 h) are related to the cell cycle regulation and basic gene expression mechanisms, whereas pathways associated with mRNA targets of downregulated miRNAs are related to inflammatory processes (4 h) or extracellular matrix (ECM) signalling (8 and 24 h).

### Extracellular vesicle proteome

Analysis of the EV proteomics data identified 493 proteins, with an average of 306 (standard deviation 11) proteins per TMT experiment. The bioinformatic and downstream analyses were conducted on the 214 common proteins that were observed across all TMT experiments (Supplementary Methods and Supplementary File D).

**Fig 3:**
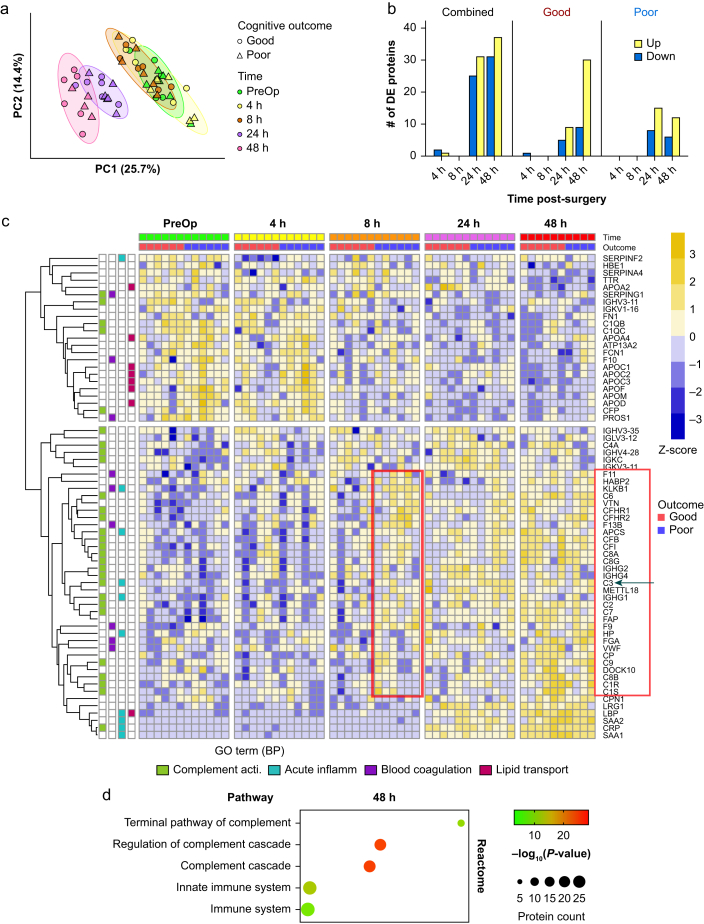
Differential expression (DE) of proteins in circulating extracellular vesicles (EVs). (a) Principal component (PC) analysis of the EV proteome (normalised abundancies of 214 proteins consistently expressed in all samples). (b) Number of DE proteins in the combined, good, and poor outcome groups. (c) Hierarchical clustering analysis of the normalised abundances of the 66 DE proteins. Data are presented for each patient across all timepoints and separately for each cognitive outcome group. The proteins showing different protein abundances at the 8-h timepoint between groups (mostly complement system) are enclosed in red. Green arrow indicates the central complement component C3. Each protein is associated with its corresponding Gene Ontology (GO) term: biological function (BP; bottom). (d) Pathway enrichment analysis using the merged list of 66 DE proteins from the good and poor outcome groups at the 48-h timepoint. Presented are the results from the David-based search in the Reactome database visualizing the top significantly (false discovery rate ≤0.05) enriched pathways. The DE protein list from the 24-h timepoint generated a nearly identical bubble plot (results not shown). PreOp, preoperative.

**Fig 4:**
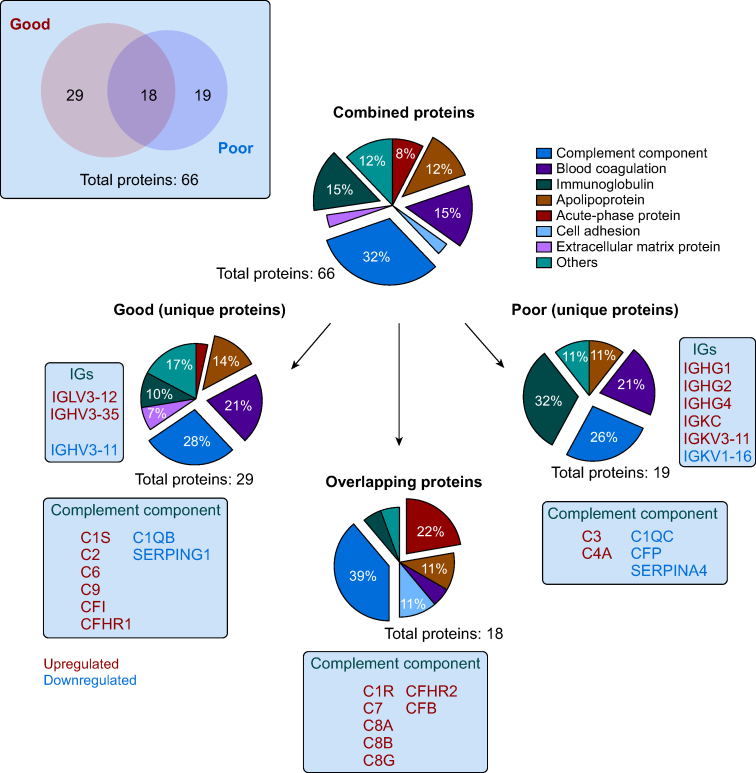
Venn diagram and protein classification analysis of the 66 differentially expressed proteins. Among the 66 differentially expressed proteins, seven main classes of proteins were identified (see legend). The complement component was the class with the highest percentage of proteins (32%), followed by immunoglobulin-related proteins (15%), blood coagulation proteins (15%), apolipoproteins (12%), and acute-phase proteins (8%). Analysis of the set of proteins unique for the poor group showed relatively higher abundance of immunoglobulin-related proteins compared to the good group (32% *vs* 10%). The complement component was the most represented term within the unique and overlapping proteins in the two groups. Enclosed rectangles indicate the name and the vector of the regulation of the proteins included in the immunoglobulin (IG) and complement component terms.

PCA revealed two unique EV proteome signature clusters at 24 and 48 h after surgery independent of the postoperative cognitive outcome groups (Fig. 3a). Differential protein abundance analysis in the combined group revealed 56 (25 down/31 up) and 68 (31/37) differentially expressed proteins at 24 and 48 h, respectively (Fig. 3b). A similar temporal pattern was observed when analysing the proteins per group, with most differentially expressed proteins found at the same postoperative timepoints (Fig. 3b). To analyse the postoperative temporal variations for the good and poor outcome groups, we selected all differentially expressed proteins observed between the two groups at each timepoint, regardless of the directionality of the change (decrease or increase), and conducted hierarchical clustering analysis using the normalised abundance data of the entire set. This set of differentially expressed proteins comprised 66 proteins (23 downregulated and 43 upregulated) at either 24 or 48 h after surgery (Supplementary File D). This clustering analysis revealed two clusters of differentially expressed proteins (Fig. 3d). Within the downregulated cluster, both outcome groups displayed a similar temporal pattern characterized by a gradual decrease in protein abundances from 8 to 48 h after surgery. Functionally, proteins in this cluster belong to the complement system, blood coagulation, and lipid transport groups, as indicated by the Gene Ontology (GO) enrichment analysis. Conversely, within the upregulated cluster, both groups exhibited a gradual increase in protein abundance, most prominently observed at 48-h postoperatively. The GO enrichment analysis of this cluster demonstrated the presence of proteins involved in complement system activation. The temporal pattern of changes in complement protein abundance differed depending on the group: the poor outcome group showed an earlier (8 h *vs* 24 h) increase in the abundance of proteins related to the complement system compared to the good outcome group (Fig. 3d). Irrespective of neurocognitive outcome, a group of proteins (C-reactive protein [CRP], serum amyloid A1 [SAA1], serum amyloid A2 [SAA2], and lipopolysaccharide-binding protein [LBP]) associated with the ‘acute inflammation’ term exhibited a marked increase at 24 and 48 h after surgery (Fig. 3d). Further analysis of 66 differentially expressed proteins showed that only 18 overlapped between the two groups, where 22% were classified as acute phase proteins and 39% as complement components (Fig. 4 and Supplementary File D). Functional analysis of the unique and overlapping differentially expressed proteins indicated that the same classes of proteins were evenly represented within the different sets of proteins, suggesting that surgery affects the same pathways regardless of postoperative neurocognitive outcome. The most striking difference between outcome groups involved immunoglobulin-related proteins, which were more abundant in the poor outcome group, although their number was generally low (3 in the good *vs* 6 in the poor group; Fig. 4 and Supplementary File D).

Pathway analysis for the combined group of 66 differentially expressed proteins from the good and poor outcome groups at 48 h confirmed the prevalence of mostly complement system-related pathways (Fig. 3d and Supplementary File E).

#### Surgery increases the concentrations of extracellular vesicle-associated complement proteins

To further delve into the longitudinal differences observed among the EV complement-associated proteins between the two neurocognitive outcomes, we compared temporal changes in protein abundance between groups. With few exceptions (Complement C1q subcomponent subunit B(C1QB), Complement C1q subcomponent subunit C (C1QC), and Properdin (CFP)), the poor outcome group exhibited significant upregulation of most of the detected complement proteins at 8 h after surgery, with most of these proteins showing a persistent increase at 48 h. In contrast, in the good outcome group, this response was not as clear, although a trend towards an increase was observed in some proteins at 48 h (Supplementary Fig. 5). Among these proteins, C3 was the complement component with the strongest differences between groups (Supplementary Fig. 5 and 5a, left panel).

### Impact of surgery on total C3 in serum and CSF

**Fig 5:**
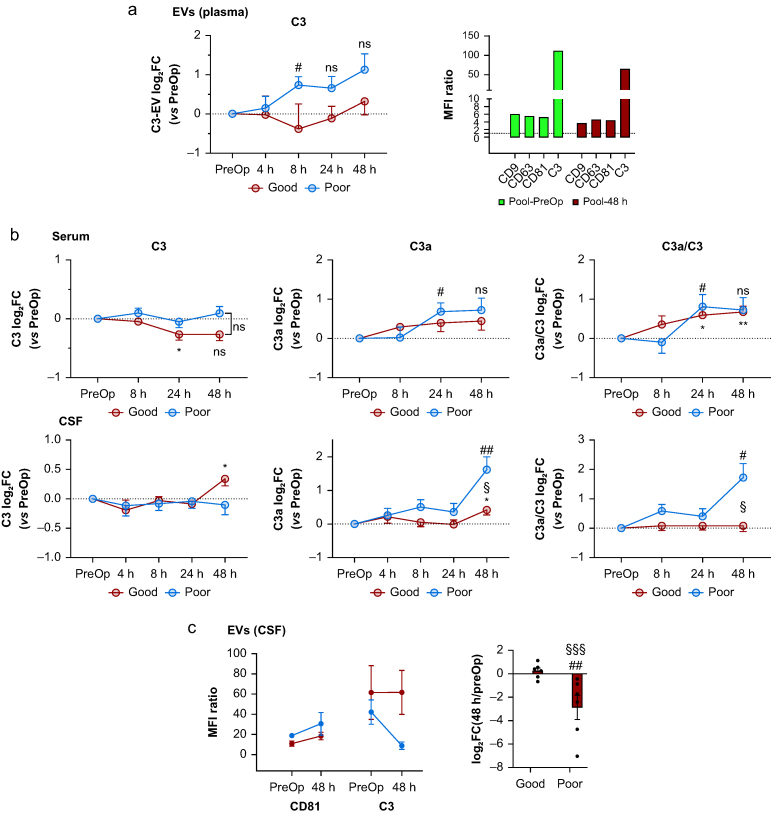
Effect of surgery on total and extracellular vesicle (EV)-associated C3 in serum and cerebrospinal fluid (CSF). (a) Left panel: log_2_ fold changes (log_2_FC) of the relative C3 concentrations determined by mass spectrometry from plasma-derived EVs isolated from the good and poor neurocognitive outcome groups relative to corresponding preoperative (PreOp) values. Patients in the poor cognitive outcome group (poor) showed an early and persisting increase in the abundance of C3 in plasma-derived EVs (two-way analysis of variance with repeated measures: multiple comparison; uncorrected Fisher's least significant difference [LSD]): poor (#, *P*=0.0185, ns <0.1). Right panel: confirmation of the C3 association with plasma EVs. Latex beads were coated with anti-human CD9, incubated with Exo-EVs from plasma, and analysed for the presence of CD9, CD63, CD81, and C3 by flow cytometry for pooled EVs from three patients (two from the good cognitive outcome group and one from the poor cognitive outcome group) at the PreOp and 48-h timepoints. Mean Fluorescence Intensity (MFI) ratio is the ratio of mean fluorescence intensity of capture antibody against isotype control. (b) Enzyme-linked immunosorbent assay-based quantification of C3, C3a, and resulting C3a-to-C3 ratio in the serum and CSF in both cognitive outcome groups. Data are presented as log_2_FC relative to PreOp values. Statistical differences were tested by two-way analysis of variance with repeated measures followed by multiple comparison analysis by uncorrected Fisher's LSD: good ∗*P*<0.05, ∗∗ *P*<0.01 (*vs* PreOp); poor #*P*<0.05, ##*P*<0.01 (*vs* PreOp); good *vs* poor, §*P*<0.05; ns, *P*<0.1. (c) Left panel: C3 log_2_FC relative to PreOp values from the flow cytometry experiment on CSF-derived EVs from good and poor groups at 48 h after surgery. Two-way analysis of variance with repeated measures: time: time × outcome: ∗*P*=0.0156. Multiple comparison (uncorrected Fisher's LSD): poor (##*P*=0.0035) and good *vs* poor (§§§*P*=0.0005). Right panel: results from the flow cytometry analysis of CD81 and C3 from the CSF-derived EVs (EVs CSF) of patients from both outcome groups.

To obtain insights into the nature of the C3–EV association, we applied a flow cytometry-based assay of C3 on two pools of Exo-EVs from three patients at preoperative and 48 h postoperative timepoints, which were additionally immunoaffinity purified using the EV marker CD9 antibody-conjugated beads. A strong C3 signal was detected at both timepoints, indicating a robust surface C3–EV association (Fig. 5a, right panel). To further understand the relevance of this post-surgical C3–EV association, we measured total concentrations of circulating C3 and its proteolytic fragment C3a in the serum of all patients, and computed the C3a-to-C3 ratio, an indicator of complement activation.^14^ Despite mild differences in the postoperative (24 and 48 h) impact of surgery on C3 concentrations in the good outcome group, the overall systemic activation of the complement system triggered by surgery was similar between groups (Fig. 5b, upper panel).

In recent studies, dysregulated C3 concentrations in CSF have been linked to various neurodegenerative disorders, such as Alzheimer's disease (AD) and dementia, via activation of C3a/C3Ra inflammatory signalling.^15^ Therefore, we tested whether surgery might affect the CSF complement system differently depending on postoperative cognitive outcome by measuring total C3 and C3a concentrations in CSF across all timepoints. The average C3a-to-C3 ratio at 48 h in CSF was about four-fold higher compared with the average preoperative value in the poor group in contrast to no change in the good group (Fig. 5b, lower panel).

We also investigated whether the C3–EV association was present in CSF-derived EVs of these patients. As in plasma-derived EVs, C3 was highly associated with CSF-derived EVs (Fig. 5c, left panel). There was a strikingly different temporal association pattern between the two outcome groups: in the good outcome group, the preoperative C3 signal remained stable in the CSF-derived EVs at 48 h after surgery, while in the poor outcome group there was a large reduction in the C3–EV signal (Fig. 5c, right panel).

## Discussion

We described the temporal impact of surgery on circulating EV proteome and miRNAome in humans, which adds a new dimension to previous studies based on the same clinical material.^4^^,^^5^ We established temporal changes in the composition of constituent molecules of circulating EVs after surgery: miRNAs were differentially expressed mostly at early timepoints while changes in the EV proteome occurred at later times. Secondly, modifications observed in the miRNAome and proteome (mostly complement proteins) of plasma-derived EV were primarily associated with poor neurocognitive outcome.

Although it is difficult to predict the target tissue(s) of circulating EVs, the fact that most differentially expressed miRNAs were identified in the EVs of patients with neurocognitive decline after surgery suggests potential functional impact on the brain. Analysis of enriched pathways that involve mRNA targets of the three upregulated miRNAs at 4 h points to downregulation (assuming the negative impact of miRNAs on mRNA translation) of pathways that stimulate proliferative and growth processes. One of these miRNAs, miR-127-3p, was reported to inhibit MDM2, an E3 ubiquitin ligase-negative regulator of p53, an important tumour suppressor.^16^ In the central nervous system (CNS), overexpression of p53 can result in accelerated neuronal damage as observed for different brain pathologies, such as ischaemia, epilepsy, and neurodegeneration.^17^^,^^18^ Interestingly, using the same set of samples, we previously found increased serum concentrations of neurofilament light protein, which also signal potential neuronal damage.^5^

Several critical signalling regulators and their downstream targets are among the prominent mRNA targets for another upregulated miRNA, miR-193a-5p. The regulatory subunit of phosphoinositide 3-kinase (PI3K), phosphoinositide-3-kinase regulatory subunit 3 (PIK3R3), and its downstream protein kinase mammalian target of rapamycin (mTOR) are part of the PI3K–protein kinase B (Akt)–mTOR axis which are considered essential for learning, memory, and synaptic plasticity in the hippocampus.^19^ Using a mouse model of surgery-induced cognitive decline, we previously showed that orthopaedic surgery affects hippocampal synaptic transmission and plasticity.^20^

Simultaneously with the decreased activity of proliferative pathways, downregulation of three miRNAs including miR-150-5p at 4 h after surgery in the poor group could result in increased activity of various inflammatory processes including cytokine signalling. Another miRNA significantly downregulated at 8 and 24 h, miR-29c-3p, inhibits translation of many mRNAs involved in the regulation of ECM proteins, a process closely linked to wound healing.^21^ The same miRNA was also downregulated in circulating EVs of the mice subjected to orthopaedic surgery.^9^

The miRNA miR-342-5p is reduced in total plasma^22^ and plasma-derived EVs^23^ in patients with AD, and the extent of downregulation correlates with the degree of cognitive decline.^22^ The miR-342-5p concentrations were also reduced along with other miRNAs, such as miR-150-5p, miR-29b-3p, and miR-23b-3p.^23^ All four were found to be downregulated in our study (the last two though missed the cut-off of significance). However, the biological effects of reduced miR-342-5p in circulating EVs on the brain remain elusive despite reports on brain-expressed miR-342-5p involvement in AD axonopathy^24^ and in the regulation of synaptic genes.^22^

Despite careful selection of potential mRNA targets for the differentially expressed miRNAs and subsequent analysis of the statistically significant enriched biological pathways, conclusions regarding the potential regulatory impact of EV miRNAs on target tissues remain hypothetical and require experimental validation. The same applies to potential roles of differentially expressed miRNAs in the regulation of processes related to cognitive function in the brain.

### Proteomics

We observed that surgery triggered a similar temporal EV proteome signature in both groups of patients. Surgery mostly modulated proteins related to activation of the innate immune system, such as acute phase proteins and proteins involved in the coagulation cascade and complement system activation, suggesting a contribution of circulating EVs to the systemic inflammatory response triggered by surgery. This is consistent with possible upregulation of inflammatory pathways in EV target tissues because of the downregulation of miR-150-5p in the poor group, albeit at earlier timepoints.

The temporal pattern of complement protein expression between neurocognitive outcome groups was different. Thus, plasma-derived EVs from the poor neurocognitive outcome group showed an earlier and more profound increase in most of the identified complement components compared with their counterparts in the good neurocognitive outcome group. This was particularly relevant for the complement component C3, the central protein in the complement system which is processed into C3a and C3b fragments, an essential step for the initiation and activation of all canonical complement pathways.^15^ However, such temporal and group-specific C3–EV upregulation patterns do not correlate either with corresponding total serum C3 concentrations or with the C3a-to-C3 ratio, that is, complement activation. This suggests a specific regulatory role for C3-loaded EVs in communication with target tissues with no influence on circulating concentrations of total C3.

Our previous findings concerning the short-lasting and early disruption of the blood–brain barrier observed in these patients during the first 8 h after surgery, more pronounced in the poor neurocognitive group,^4^^,^^5^ prompt us to hypothesise that C3-loaded plasma-derived EVs can be directly transferred to the brain through the blood–brain barrier where the C3 can then exert its modulatory function. We observed a significant increase in total C3 concentrations in the CSF of patients from the good neurocognitive group at 48 h after surgery. However, C3 is also locally produced by astrocytes within the CNS,^25^ therefore the origin of total C3 detected in the CSF is not clear. Nevertheless, while we did not detect any variation in the CSF total C3 concentrations in the poor outcome group, we observed a four-fold increase in C3a concentrations (and the C3a-to-C3 ratio). This increase suggests profound complement system activation in the brain exclusively in patients with long-term cognitive decline. Similar activation of complement signalling was shown to be associated with postoperative cognitive dysfunction in an animal model of orthopaedic surgery.^26^

Increased C3–EV plasma concentrations have been described for several pathological conditions, such as multiple sclerosis, obesity, and rheumatoid arthritis.^27^, ^28^, ^29^ Moreover, in a cell model of pulmonary hypertension, C3–EVs were shown to drive a proinflammatory and metabolic reprogramming of macrophages.^30^ These and other studies suggest complex complement–EV interactions, with a potential impact on local and systemic inflammation.^31^, ^32^, ^33^

In the CNS, complement proteins have been shown to participate in synapse pruning, neurogenesis, and neuronal survival in both developing and mature brains.^15^^,^^34^ Microglia complement receptor 3, expressed on the surface of the microglia, and C3, enriched in synaptic compartments, interact to mediate engulfment of synaptic elements undergoing active pruning,^35^ an essential mechanism for forgetting of encoded memories in adult brain.^36^ Synapse loss, neuroinflammation, complement upregulation, and cognitive decline are major events described in a great variety of neurodegenerative diseases, with special relevance in AD.^37^ Increased concentrations and activation of C3 in the CSF from patients with AD correlate with tau pathology and disease progression.^38^ Moreover, deletion of C3 in mouse models of neuropathy (PS2APP and APP/PS) and tauopathy (TauP301S) rescued synapse loss and ameliorated neurone loss and brain atrophy, improving neurophysiological and behavioural measurements.^38^^,^^39^ Although more functional studies are required to understand the relevance of the C3–EV association in normal physiology and pathology, the differential C3–EV association patterns observed between patients with good and poor postoperative cognitive outcomes in plasma- and CSF-derived EVs suggest a potential mechanism by which peripheral tissue injury might impact higher brain functions.

Our flow cytometry data suggest that the association of complement factors with circulating EVs and CSF occurs, at least for C3 protein, on the EV surface rather than inside EVs. This is in line with studies showing association of EVs with the ECM and different plasma proteins, and describing the surface interactome of EVs (protein corona) and its potential functional implications in health and disease.^40^, ^41^, ^42^, ^43^ Although more functional studies are required to understand the relevance of this association in normal physiology and pathology, the fact that we were able to observe differential C3–EV association patterns between patients with good and poor postoperative cognitive outcomes in plasma- and CSF-derived EVs suggests a novel mechanism by which peripheral tissue injury could acutely impact higher brain functions.

A major limitation of the study is the relatively low number of patients included in each of the cognitive outcome groups due to the complexity of the analysis. This was partially compensated for by the repeated-measure design in which values for each patient were compared to their preoperative value. However, the findings of this study should be considered preliminary and require validation in a larger surgical patient cohort.

In conclusion, surgical trauma reprograms expression of plasma extracellular vesicle-associated miRNAs and proteins. Most of these changes were encountered exclusively in patients experiencing postoperative neurocognitive decline. The differential experession of miRNA might affect proliferation, inflammatory, and extracellular matrix biological pathways in their target tissues, including the brain. The temporal changes in protein expression were most evident for members of the complement system and its major constituent, C3. While systemic complement activation was detected in both cognitive outcome groups, we detected a CSF complement activation pattern among patients with postoperative neurocognitive decline.

## Authors’ contributions

Conception and design: MGG, SM, LIE

Sample and data acquisition: MGG, AE, ME, MD, AW, JO, AV

Analysis and interpretation of the data: MGG, SM, ME

Funding acquisition: LIE

Drafting the manuscript: MGG, SM

Reading and revision of the manuscript: all authors

## Declarations of interest

SG has a patent on B-cell-targeted exosomes in immune therapy and is part of the Scientific Advisory Board of Anjarium Biosciences. The other authors declare no competing interests.

## Funding

LIE has received funding for the study from the following sources: Research Council Medicine, Stockholm, Sweden: Grant nr 2023-02000 and 2020-01485. Region Stockholm Council - ALF project grants, Stockholm, Sweden: Grant nr FoUI-985353 and FoUI-955054. Brain Foundation, Stockholm, Sweden: Fo2023-0150 and 2021-0066.
