# Supplementary material for: Changes in circulating extracellular vesicle cargo are associated with cognitive decline after major surgery: an observational case–control study
Source: Br J Anaesth. 2024 Oct 18;134(6):1683–95. doi: 10.1016/j.bja.2024.07.040 (PMC12106869; doi:10.1016/j.bja.2024.07.040)
Supplement: Multimedia component 1 [file mmc1.zip › Supplementary Methods_BJA-D-24-00112.docx]

**Modification of circulating extracellular vesicles’ cargo is associated with cognitive decline after major surgery**

**Souren Mkrtchian, Maria Eldh, Anette Ebberyd, Susanne Gabrielsson, Ákos Végvári, Sven-Erik Ricksten, Mattias Danielson, Jonathan Oras, Andreas Wiklund, Lars I Eriksson, Marta Gómez-Galán**

**Methods**

*Patients and study design*

This study is a follow-up to a longitudinal prospective observational case-control study (NEUPORT) ^1, 2^, conducted on 34 patients between September 2014 and March 2016 at the Karolinska University Hospital, Stockholm (n=14) and Sahlgrenska University Hospital, Mölndal (n=20). The NEUPORT study was approved by the Regional Ethics Committee in Stockholm, Sweden (Dnr 2013/2297–31/4 and 2014/834–32) and registered at www.clinicaltrials.gov; identifier: NCT02759965.

*Neurocognitive testing and group selection*

Patients’ preoperative (1-2 weeks before surgery) and postoperative (3-5 days and 3 months after surgery) neurocognitive capacities were evaluated using the International Study of Postoperative Cognitive Dysfunction (ISPOCD) test battery which included seven test variables ^3^.

In brief, this test battery measure cognitive performance using four different tests, providing seven variables for analysis, *i.e.*, the cumulative number of words recalled in three trials and the number of words at delayed recall from the visual verbal learning test (VLT), the time (seconds) and number of errors (n) in part C of the concept shifting test (CST), the time (seconds) and number of errors (n) scores from the third part of the Stroop color word interference test (SCWT) and the number of correct answers (n) from the Letter digit coding test (LDC). The duration of a test session was approximately 30 min ^1^.

Changes in cognitive performance were calculated for each of the 7 test variables and corrected for the learning effect using data from an age-matched control group that was tested using the same battery and with the same intervals. To quantify the change in cognitive capacities from preoperative to postoperative tests, a z-score calculated for each variable was combined into a composite z-score. The patients with a composite z-score ≥ 1 at 3 months post-surgery were assigned to the poor neurocognitive outcome group while those with a composite z-score < 1 were included in the good neurocognitive outcome group. Based on this categorization, six patients from the good outcome group and six patients from the poor outcome group were selected for downstream analyses. The demographic data for all 12 patients included in the study is presented in Table 1.

*Anaesthesia, blood, and CSF sample collection*

Briefly, after placement of an intrathecal catheter, the surgical procedure was performed under spinal anaesthesia supplemented by light sedation. The intrathecal catheter was left in place for 48-hours to allow serial CSF sampling. CSF (5 ml) and blood (20 ml) were collected preoperatively and at 4-, 8-, 24-, and 48-hours after skin incision. Serum and plasma were prepared as previously described ^1^, aliquoted, and stored at -80°C for subsequent analysis.

*Extracellular Vesicle Isolation Methods*

Due to limiting plasma material, EVs were isolated from the patients’ blood plasma using two methodological approaches depending on the downstream analysis: i) a commercial precipitation method and ii) size-exclusion chromatography.

Precipitation: ExoQuick ULTRA. Isolation of EVs was performed with ExoQuick ULTRA (System Biosciences Inc., Mountain View, CA) according to manufacturer’s protocol and as recently described in ^4^. In brief, an initial 250 µl of plasma was incubated with 67 µl of the commercial ExoQuick and, after a serial of washing steps, the EVs were finally eluted in approximately 500 µl of the commercial elution buffer. An aliquot of 50 µl was stored at 4°C for further validation studies (electron microscopy and NTA) while the rest was stored at -80°C until further downstream analysis (i.e., proteomics, western blot, flow cytometry). The depleted EV (dEV) fraction was also collected and stored at -80°C.

Size-exclusion chromatography: Izon 70. The 70 nm qEVoriginal size exclusion columns (Izon 70) (Izon Science Limited, Christchurch, New Zealand).) were used according to the manufacturer’s protocol and as described in ^5^. In brief, 1 ml of plasma samples were centrifuged at 1500 x *g* for 10 min and supernatants were re-centrifuged again at 10,000 x *g* for 10 min. 0.5 ml of the resulting cleared plasma was loaded onto the column and eluted with PBS. The first fraction (~2.5 ml, default buffer volume) was discarded and the next four fractions (with 0.4 ml increments), containing most of the EV-sized particles according to the manufacturer’s protocol, were collected. The procedure was repeated for the remaining 0.5 ml of cleared plasma from the same patient. The final EV fractions were pooled and subsequently concentrated to ~250 µl volume using Amicon Ultra-4 centrifugal filters (cut-off 30 kDa, Merck Millipore Ltd.). An aliquot of 50 µl was stored at 4°C for further validation studies (electron microscopy and NTA) while the rest was stored at -80°C until further down-stream analysis (i.e., RNA isolation, western blot, and flow cytometry).

*Nanoparticle Tracking Analysis*

The particle concentration and size distribution of plasma-derived EV isolates were measured using the Nanosight LM10HSB system. The system is equipped with a 405 nm laser running NTA 2.3 analytical software package. The samples were diluted in filtered PBS (1:100) to a range of 5 × 10^9^ - 1 × 10^12^ particles/ml and analysed with camera level 13 and detection threshold 3 with a syringe pump speed of 50. For each sample, four consecutive videos, 30 each, were recorded.

*Negative stain transmission electron microscopy*

Electron microscopy analysis was conducted at the electron microscopy unit EMil (Karolinska Institute) as described previously (^4^). Transmission electron microscopy (TEM) imaging was done using Hitachi HT7700 (Hitachi High-technologies) transmission electron microscope operated at 100 kV equipped with a 2kx2k Veleta CCD camera (Olympus Soft Imaging System).

*Small RNA Sequencing*

RNA isolation. Total RNA was extracted from iZon70-isolated EVs (Izon-EVs) using the Exosomal RNA isolation kit from Norgen Biotek Corp. (Nordic BioSite AB, Täby, Sweden).

Library preparation. RNA quality was controlled with Agilent Bioanalyzer 2100 (Agilent, Palo Alto, CA) equipped with a small RNA chip. Small RNA libraries were constructed using the NEXTFLEX® Small RNA-Seq Kit v3 (Bioo Scientic Corp., Austin, Texas, USA) according to the manufacturer’s protocol ^4^.

Sequencing. All samples were pooled in equimolar ratio and sequenced on the Illumina NextSeq 500/550 high output flowcell, with a 75- cycle kit, single read for 84 cycles plus 7 cycles for the index read.

Library construction, quality control, and sequencing were conducted at the Genomics Core Facility of the Institute of Molecular Biology GmbH (IMB), Mainz, Germany.

Data processing. The quality of the raw sequencing reads was assessed by FastQC v.0.12 before and after adapter sequences removal and size-selection for 14-34 base-long inserts using cutadapt v 4.1 (‘-u 4 -a NNNNTGGAATTCTCGGGTGCCAAGG -m 14 -M 34’). The resulting trimmed reads were aligned against the human genome (GRCh38/hg38) using Bowtie v.1.3.1. Genome-aligned reads in SAM were converted into sorted BAM files with Samtools v 1.9, and read counts were summarized per mature miRNA using featureCounts (subread v 2.0.0) and miRNA annotation from miRbase v.23 (https://mirbase.org/). The final matrix of read counts was posteriorly used for down-stream data analysis.

The computations were enabled by resources provided by the National Academic Infrastructure for Supercomputing in Sweden (NAISS) and the Swedish National Infrastructure for Computing (SNIC) at Uppsala (Sweden) partially funded by the Swedish Research Council through grant agreements no. 2022-06725 and no. 2018-05973.

Data analysis. Differential expression (DE) analysis between each post-operative timepoint (4, 8, 24, 48 h) and the preoperative baseline was carried out using the online Bioconductor R package DEBrowser ^6^ (https://debrowser.umassmed.edu/). The DE analysis was carried out in the combined group (no discrimination between post-operative neurocognitive outcomes) or separately in the groups of patients with good or poor post-operative cognitive outcomes. In the combined group we included all the samples (n=59) categorized by timepoints (n=12/timepoint except for the 48 h timepoint in which n=11). In the good and poor groups case the number of samples per timepoint was reduced to half (n=6/timepoint and condition except for the 48 h timepoint in the poor group in which n=5).

To remove low-expression miRNAs from the original read counts matrix, counts per million (CPM, raw counts divided by the library size and multiplied by one million) were calculated. miRNAs with less than 10 CPM in the smallest group sample size (n=11 for the combined group and n=5 for the good/poor groups) were removed from the original matrix. Given the difference in group sizes between the combined and good/poor analysis, the filtering resulted in the generation of two different (but similar) filtered read count matrices (see Results and Supplementary file A). The downstream DE analysis was applied for both filtered read count matrices, with no differences in terms of applied parameters/models.

The filtered count data was subsequently normalized by the Trimmed Mean of M-values (TMM) and analysed by the embedded *Limma-Voom* R package in which the *voom* transformation was applied to the normalized and filtered count data. miRNAs with P value ≤ 0.05 and false discovery rate (FDR) ≤ 0.1 were considered statistically significant and referred to as DE miRNAs.

Principal component analysis (PCA) was generated using Qlucore Omics Explorer 3.2 (Qlucore, Lund, Sweden) using log2-normalized CPM expression values from the filtered count data.

Identification of potential target mRNAs for each DE-miRNA was based on the stringent approach were only mRNAs that were experimentally confirmed (typically by gene reporter, PCR, western blot assays) to interact with the miRNA in question were included in the further pathway analysis. Briefly, the identification involved searches in three distinct databases: i) ENCORI (https://rnasysu.com/encori) ^7^, ii) )TarBase v.8 (https://dianalab.e-ce.uth.gr/html/diana/web/index.php?r=tarbasev8/index) ^8^ and iii) miRTargetLink 2.0 (https://ccb-compute.cs.uni-saarland.de/mirtargetlink2) ^9^. In addition, literature mining was used to manually select potential targets by applying the same stringent criteria. The resulting lists of target mRNAs as produced by each query were merged to obtain a definitive list of target mRNA for each DE miRNA.

Pathway enrichment analysis was consequently conducted for each timepoint using the mRNA lists where the mRNA targets were combined depending on the directionality (up or down) of the miRNA differential expression. We initially tested 4 different online tools: i) David (https://david.ncifcrf.gov), ii) Enrichr-KG (https://maayanlab.cloud/enrichr-kg), iii) WebGestalt (https://www.webgestalt.org/) and iv) gProfiler (https://biit.cs.ut.ee/gprofiler/gost). However, as all of them generated similar results we selected David and gProfiler for the final analysis as having the most updated KEGG/Reactome/WikiPathways and Gene Ontology (GO) databases. Detailed enriched pathway data from Reactome, WikiPathways and KEGG databases are presented in Supplementary file C.

*miRNA qPCR*

Validation of miRNA-seq results was accomplished by qPCR of selected DE miRNAs using TaqMan® Advanced miRNA Assays (Applied Biosystems, ThermoFisher Scientific). The following TaqMan® Advanced miRNA Assays: hsa-miR-152-3p (assay ID, 477921_mir), hsa-miR-423-5p (478090_mir), hsa-miR-342-5p (478044_mir), hsa-miR-193a-5p (002281_mir) were used. Data were normalized to hsa-let-7f-5p and hsa-miR-30c-5p, which were chosen as the housekeeping miRNAs based on the analysis of expression stability of several miRNAs using the RefFinder tool (https://www.heartcure.com.au/reffinder/). The relative abundance of each miRNA was estimated according to the 2^–ΔΔCt^ method.

*Proteomics*

*Liquid Chromatography-Tandem Mass Spectrometry Based Proteome Analysis*

Quantification of relative protein abundances from ExoQuick ULTRA isolated EVs (Exo-EVs) was performed using tandem mass tag (TMT) labelling multiplex approach. Sample preparation, peptide labelling with TMT mass tag reagent, and subsequent separation of labelled peptides on EASY-Spray C18 column and mass spectra acquisition on Orbitrap Q Exactive HF mass spectrometer (ThermoFisher Scientific) was performed at the Proteomics Biomedicum core facility (Karolinska Institutet, Stockholm, Sweden (https://ki.se/en/mbb/proteomics-Biomedicum).

The total 59 Exo-EV samples were organized by patient (n=12) and timepoints (PreOp, 4-, 8-, 24- and 48-hours) and the labelled peptides for each sample were loaded in a balanced manner in 6 different TMT-11 plex (TMT experiments). Therefore, in each TMT experiment a full set of samples per patient (i.e., 5 samples) and per condition (good/poor) was included. In addition, for data normalization a pool sample (i.e., internal reference), containing an equal amount of peptide/protein for each 59 samples, was included.

*Sample preparation*

Isolated EVs were dried on a vacuum concentrator (Eppendorf) and resolubilized in 25 µl of 8M urea in 50 mM Tris-HCl, pH 8.5 sonicated in the water bath for 5 min before 25 µl of 0.2% ProteaseMAX surfactant (Promega) in 10% acetonitrile (ACN) and 4x protease inhibitor cocktail (Roche) in 50 mM Tris buffer was added. Following sonication in the water bath for 5 min, 50 µl of 100 mM Tris-HCl buffer was added and sonicated using VibraCell probe (Sonics & Materials, Inc.) for 20 s with pulse 2/2 (on/off), at 20% amplitude. Protein concentration was determined by BCA assay (Pierce) and a volume corresponding to 25 µg of protein of each sample was taken and supplemented with Tris-HCl buffer up to 70 µl. A pooled sample to be used for normalization was composed combining 4.2 µg of proteins from six individual samples and prepared identically. Proteins were reduced with 7 µl of 100 mM dithiothreitol incubated at 37°C for 45 min and then alkylated with 16 µl of 100 mM chloroacetamide in the dark for 30 min. Then 1 µg of sequencing grade modified trypsin (Promega) was added to the samples and incubated for 16 h at 37°C. The digestion was stopped with 5 µl cc. formic acid (FA), incubating the solutions at RT for 5 min. The sample was cleaned on a C18 Hypersep plate with 40 µl bed volume (Thermo Fisher Scientific), and dried using a vacuum concentrator (Eppendorf). Peptides, equivalent of 25 µg protein, were dissolved in 70 µl of 50 mM TEAB, pH 7.1, and labeled with TMT-11plex mass tag reagent kit (Thermo Fisher Scientific) adding 100 µg reagent in 30 µl dry ACN in a scrambled order and incubated at RT for 2 h. The reaction was stopped by the addition of hydroxylamine to a concentration of 0.5% and incubation at RT for 15 min before samples were combined and cleaned on a C-18 HyperSep plate with 40 µl bed volume (Thermo Fisher Scientific).

*Liquid Chromatography-Tandem Mass Spectrometry Data Acquisition*

The reconstituted peptides in solvent A (0.1% FA in 2% ACN) were separated on a 50 cm long EASY-Spray C18 column (Thermo Fisher Scientific) connected to an Ultimate 3000 nano-HPLC (ThermoFisher Scientific) using a gradient from 2-26% of solvent B (98% AcN, 0.1% FA) in 90 min and up to 95% of solvent B in 5 min at a flow rate of 300 nL/min. Mass spectra were acquired on an Orbitrap Q Exactive HF mass spectrometer (Thermo Fisher Scientific) in *m/z* 375 to 1700 at the resolution of R=120,000 (at *m/z* 200) for full mass, followed by data-dependent higher energy collisional dissociation (HCD) fragmentations of 18 of the most intense precursor ions with a charge state 2+ to 7+. The tandem mass spectra were acquired with a resolution of R=60,000, targeting 2x10^5^ ions, setting quadrupole isolation width to 1.4 Th and normalized collision energy to 33.

Acquired raw data files were analysed using Proteome Discoverer v2.4 (Thermo Fisher Scientific) with Mascot Server v2.5.1 (Matrix Science Ltd., UK) search engine against human protein database (SwissProt). A maximum of two missed cleavage sites were allowed for full tryptic digestion, while setting the precursor and the fragment ion mass tolerance to 10 ppm and 0.02 Da, respectively. Carbamidomethylation of cysteine was specified as a fixed modification. Oxidation on methionine, deamidation of asparagine and glutamine, as well as acetylation of N-termini and TMT-6plex, were set as dynamic modifications. Initial search results were filtered with 5% FDR using the Percolator node in Proteome Discoverer. Quantification was based on the reporter ion intensities.

*Data analysis*. Data normalization for DE analysis was conducted as described in ^10^. Sample loading (SL), Internal reference scaling (IRS), VSN normalization and batch correction (Combat) were sequentially applied to the raw dataset containing the ion intensities (abundances). The normalized-batch corrected file was used for the analysis of differential protein abundances between the preoperative and post-operative timepoints (combined group) or between the preoperative and post-operative timepoints per condition (good and poor groups) using the Bioconductor R package *limma*. Each protein was fitted into a linear model while adding patient as a random factor to adjust for patient’s inter-variation across the repeated measurements (timepoints). Proteins with log2fold change| ≥ 1.25, P value ≤ 0.05 and false discovery rate (FDR) ≤ 0.05 were considered statistically significant and referred to as differential expressed proteins (DEP).

The hierarchical clustering analysis of the normalized abundances of 66 DE proteins (Fig. 3D) is represented for each patient across all timepoints and presented separately for each cognitive outcome group. Scale representing Z-scores for each plotted protein and distance was calculated by the “clustering” (Pearson correlation) and “average” function included in the *pheatmap* package in R.

Pathway enrichment analysis was carried out using various online tools as in the miRNA analysis.

*Western blot*

EVs and dEVs were solubilized with 2% SDS and three cycles of 5 min bath ultrasound and 30 s of vortexed. Protein concentration was determined using the microBCA Protein Assay Kit (Thermo Scientific) and samples containing equal amounts of protein (5 µg) were resolved by SDS-PAGE and transferred to a PVDF membrane (Invitrogen). Depending on the secondary antibodies (HRP- or IRDye-conjugated) protein bands were detected using either enhanced chemiluminescence reagents (GE Healthcare) and ChemiDoc MO analyser (Bio-Rad) or Odyssey infrared fluorescence detection system (LI-COR, Lincoln, NE, USA).

The following primary antibody was used at a 1:500 dilution: CD63 (ab59479, Abcam).

*ELISA*

Total C3 and C3a concentrations in the serum and CSF of patients were determined using Human Sandwich (quantitative) ELISA Kit (Abcam, ab108823) and HycultBiotech (HK354) ELISA kit correspondingly as per manufacturers’ guidelines. Plates were analysed in the SpectraMax ID3 plate reader.

*Plasma and CSF-derived EV immunoaffinity capture and analysis by flow cytometry*

Bead-based flow cytometry was performed as previously described ^5^. In brief, 4-µm-diameter aldehyde/sulphate latex beads (Invitrogen) were coated with anti-human CD9 (clone HI9a, BD Biosciences) antibody under agitation overnight at RT. Next, the antibody-coated beads were blocked with 100 mM glycine for 30 minutes, washed with PBS containing 1% BSA and resuspended in PBS containing 1% BSA. A total of 1 µl (1.3 × 10^5^ beads) beads were used per staining. The isolated EV fractions from plasma, isolated by both ExoQuick Ultra (all of the sample) and Izon (2 µg EVs per µl beads), and whole CSF (250 µl) were incubated with the anti-CD9-coated beads under agitation overnight at RT. The beads-EV complexes were then washed in PBS and incubated with PE-conjugated antibodies (2 µg/ml, anti-CD9, clone HI9a; anti-CD63, clone H5C6; anti-CD81, clone 5A6; anti-C3, clone 6C9; anti-CRP, and corresponding isotype controls. All antibodies were from BioLegend, except for anti-C3 and CRP that were from LifeSpan Biosciences) for 30 min at 4°C, washed in PBS, acquired using a FACSCanto II (BDBiosciences) and analysed in Flowjo software (FlowJo LLC). Surface markers were normalised using isotype controls.

**References**

1. Danielson M, Wiklund A, Granath F, Blennow K, Mkrtchian S, Nellgard B, et al. Neuroinflammatory markers associate with cognitive decline after major surgery: Findings of an explorative study. *Ann Neurol* 2020; **87**: 370-82

2. Danielson M, Wiklund A, Granath F, Blennow K, Mkrtchian S, Nellgard B, et al. Association between cerebrospinal fluid biomarkers of neuronal injury or amyloidosis and cognitive decline after major surgery. *Br J Anaesth* 2021; **126**: 467-76

3. Moller JT, Cluitmans P, Rasmussen LS, Houx P, Rasmussen H, Canet J, et al. Long-term postoperative cognitive dysfunction in the elderly ISPOCD1 study. ISPOCD investigators. International Study of Post-Operative Cognitive Dysfunction. *Lancet* 1998; **351**: 857-61

4. Mkrtchian S, Ebberyd A, Veerman RE, Mendez-Lago M, Gabrielsson S, Eriksson LI, et al. Surgical Trauma in Mice Modifies the Content of Circulating Extracellular Vesicles. *Front Immunol* 2021; **12**: 824696

5. Veerman RE, Teeuwen L, Czarnewski P, Gucluler Akpinar G, Sandberg A, Cao X, et al. Molecular evaluation of five different isolation methods for extracellular vesicles reveals different clinical applicability and subcellular origin. *J Extracell Vesicles* 2021; **10**: e12128

6. Kucukural A, Yukselen O, Ozata DM, Moore MJ, Garber M. DEBrowser: interactive differential expression analysis and visualization tool for count data. *BMC Genomics* 2019; **20**: 6

7. Li JH, Liu S, Zhou H, Qu LH, Yang JH. starBase v2.0: decoding miRNA-ceRNA, miRNA-ncRNA and protein-RNA interaction networks from large-scale CLIP-Seq data. *Nucleic Acids Res* 2014; **42**: D92-7

8. Karagkouni D, Paraskevopoulou MD, Chatzopoulos S, Vlachos IS, Tastsoglou S, Kanellos I, et al. DIANA-TarBase v8: a decade-long collection of experimentally supported miRNA-gene interactions. *Nucleic Acids Res* 2018; **46**: D239-D45

9. Kern F, Aparicio-Puerta E, Li Y, Fehlmann T, Kehl T, Wagner V, et al. miRTargetLink 2.0-interactive miRNA target gene and target pathway networks. *Nucleic Acids Res* 2021; **49**: W409-W16

10. Plubell DL, Wilmarth PA, Zhao Y, Fenton AM, Minnier J, Reddy AP, et al. Extended Multiplexing of Tandem Mass Tags (TMT) Labeling Reveals Age and High Fat Diet Specific Proteome Changes in Mouse Epididymal Adipose Tissue. *Mol Cell Proteomics* 2017; **16**: 873-90
