# Supplementary material for: Changes in circulating extracellular vesicle cargo are associated with cognitive decline after major surgery: an observational case–control study
Source: Br J Anaesth. 2024 Oct 18;134(6):1683–95. doi: 10.1016/j.bja.2024.07.040 (PMC12106869; doi:10.1016/j.bja.2024.07.040)
Supplement: Multimedia component 1 [file mmc1.zip › Suppl-Table1.docx]

**Supplementary Table 1. qPCR validation of miRNA Seq results of selected miRNAs.**

| **miRNA** | **Post-operative timepoint** | **DE** | **Up/down/**  **unchanged (-)** | **miRNA seq** | | **miRNA qPCR** | |
| --- | --- | --- | --- | --- | --- | --- | --- |
|  |  |  |  | **Log2FC vs. PreOp** | **p.adj** | **Log2FC vs. PreOp** | **p value** |
| 193_5p | 4h | Yes | Up | 5.5 | 0.04* | 2.5 | 0.05* |
| 423-5p | 4h | Yes | Up | 1.4 | 0.04* | 0.25 | 0.49 |
| 342-5p | 4h | Yes | down | -5.4 | 0.009** | 0.49 | 0.99 |
| 222-3p | 4h | No | - | -0.03 | 0.98 | 0.38 | 0.43 |
| let-7f-5p | 4h | No | - | -0.2 | 0.76 | 0.26 | 0.34 |
| 30c-5p | 4h | No | - | -0.37 | 0.55 | -0.15 | 0.55 |
| 342-5p | 8h | Yes | down | -6.43 | 0.0004*** | 0.38 | 0.87 |
| 152-3p | 8h | Yes | down | -5.89 | 0.0008*** | -0.84 | 0.041* |
| 222-3p | 8h | No | - | 0.44 | 0.68 | 0.24 | 0.76 |
| let-7f-5p | 8h | No | - | -0.5 | 0.49 | -0.25 | 0.31 |
| 30c-5p | 8h | No | - | 0.19 | 0.81 | 0.42 | 0.24 |

DE: *Statistically differentially expressed miRNAs from the miRNA seq dataset*; Up/down/unchanged: *directionality of the observed variation in the miRNA expression relative to the pre-operative values*. Up: *increased expression*, down: *decreased expression*, unchanged: *no change in the expression*; Log2FC vs. PreOp: *Log2 foldchanges relative to preoperative values*; p.adj: *p adjusted values for RNA seq data (see Methods)*; pvalue: *p values for qPCR data (Unpaired t test with Welch's correction)*.
