# Supplementary material for: Changes in circulating extracellular vesicle cargo are associated with cognitive decline after major surgery: an observational case–control study
Source: Br J Anaesth. 2024 Oct 18;134(6):1683–95. doi: 10.1016/j.bja.2024.07.040 (PMC12106869; doi:10.1016/j.bja.2024.07.040)
Supplement: Multimedia component 1 [file mmc1.zip › Supplementary figures.pdf]

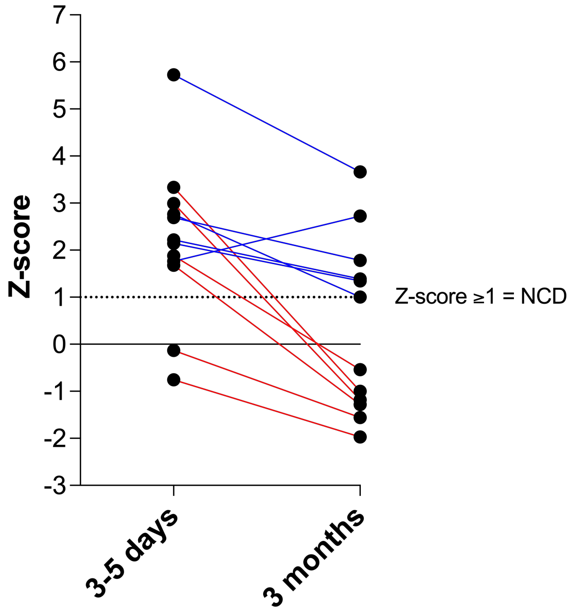

Supplementary Figure 1. Individual (n=12) neurocognitive outcome at hospital discharge (post-operative day 3-5) and at 3 months post-surgery. Those 6 patients with a composite z-score >1 at 3 monts post-surgery (i.e., patients with neurocognitive decline (NCD)) were assigned to the poor neurocognitve outcome group (blue lines) while the rest (n=6) were included in the good neurocognitve outcome group (red lines).

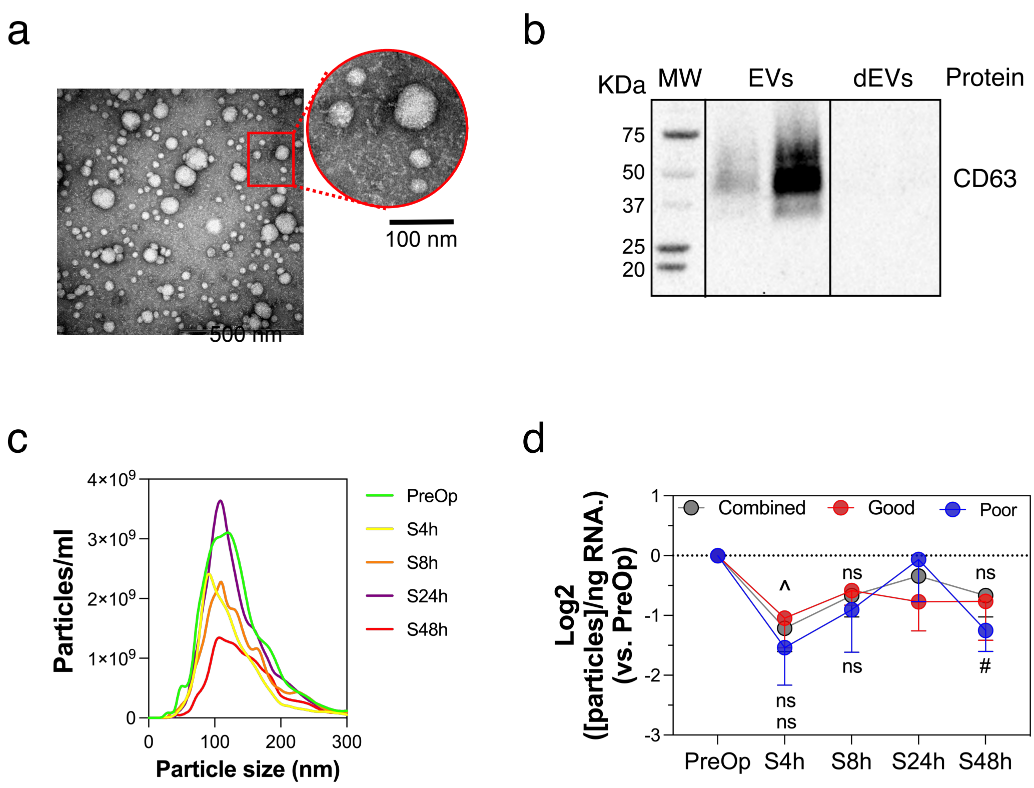

Supplementary Figure 2. Characterisation of EVs isolated by size-exclusion chromatography (iZon-EV). **a.** TEM image of the isolated particles, scale bar 500 nm. Encircle in red and magnified are representative spherical particles. **b.** Western blot identification of CD63, EV marker protein in the pre-operative plasma samples from two orthopaedic patients. dEVs, plasma depleted of EVs. **c.** Average size distribution profile of isolated particles estimated by NTA in the combined group. **d.** Log2 transformed normalized particle concentration (particle/ml) by ng of isolated RNA from the same plasma-derived EVs. Surgery decreases the number of normalised particle concentration in a time dependent manner, with no differences observed between the two groups (good vs poor) (two-way ANOVA: time: ***p<0.008). Multiple comparison relative to corresponding preoperative values (PreOp): combined (^, $p < 0.05$), poor (#, $p < 0.05$), ns: $p < 0.1$).

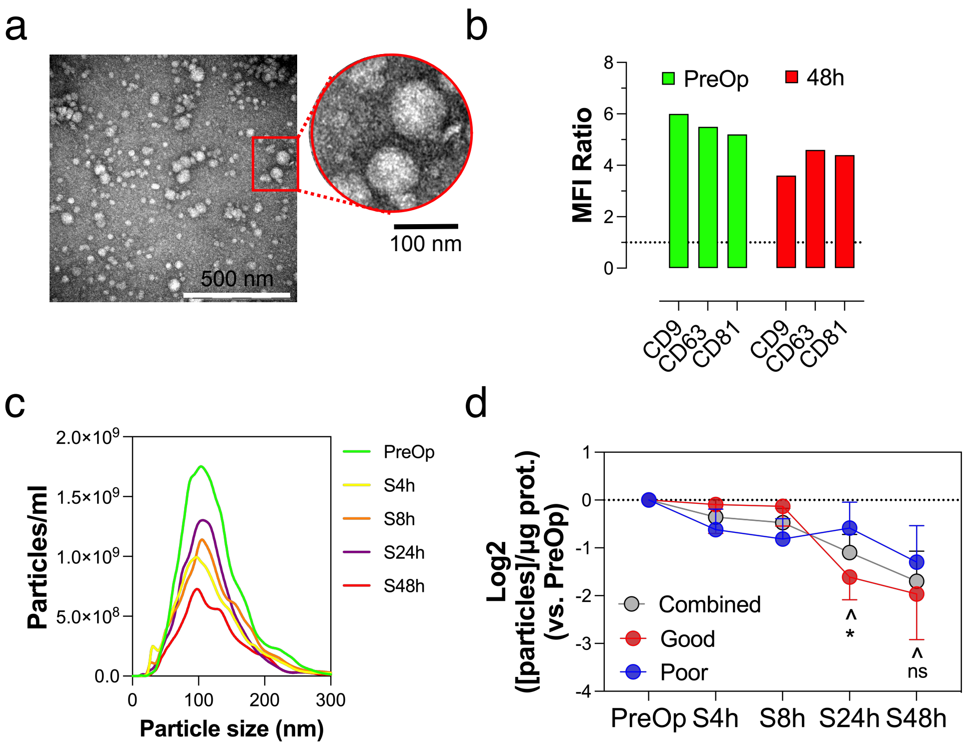

Supplementary Figure 3. Characterisation of EVs isolated by ExoQuick ULTRA (Exo-EV). a. TEM image of the isolated particles, scale bar 500 nm. Encircled in red and magnified are representative spherical particles. **b.** Flow cytometry-based validation of EV association with CD9, CD63, and CD81 EV marker proteins in pre-operative (PreOp) and 48-hours plasma samples from a pool of three patients (see Complementary Methods). **c.** Average size distribution profile of isolated particles estimated by NTA in the combined group. No differences were observed between the groups. **d.** Log2 transformed normalized particle concentration (particle/ml) by ug of total protein isolated from the same plasma-derived EVs. Surgery decreases the number of normalised particle concentration in a time dependent manner, with no differences observed between the two groups (good vs poor) (two-way ANOVA with repeated measures: time: ***p<0.0013, outcome: p = 0.88, time x outcome: p = 0.8226). Multiple comparison relative to corresponding PreOp values: combined (^^, p = 0.01), poor (#, p = 0.0201), ns, p < 0.1, for combined, good, and poor groups respectively).

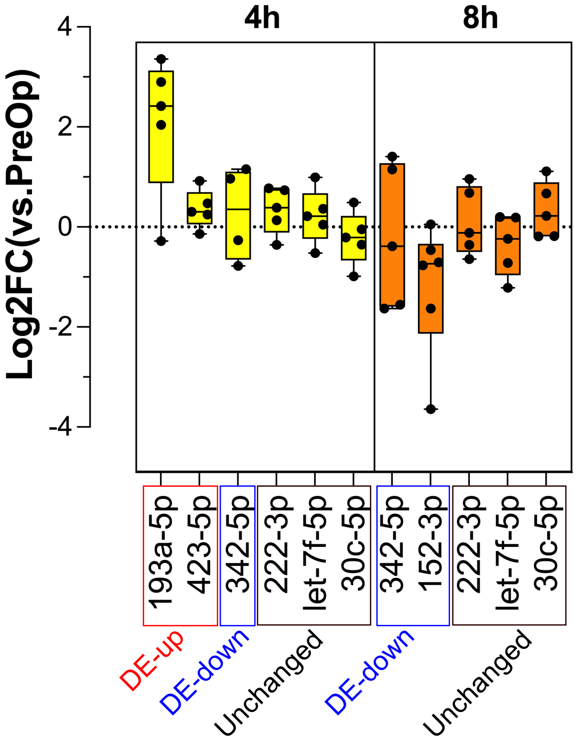

Supplementary Figure 4. qPCR Validation of miRNA-seq results using qPCR analysis of selected DE (up and down-regulated) and unregulated (unchanged) miRNAs (see Supplementary Methods and Supplementary Table 1)**.**

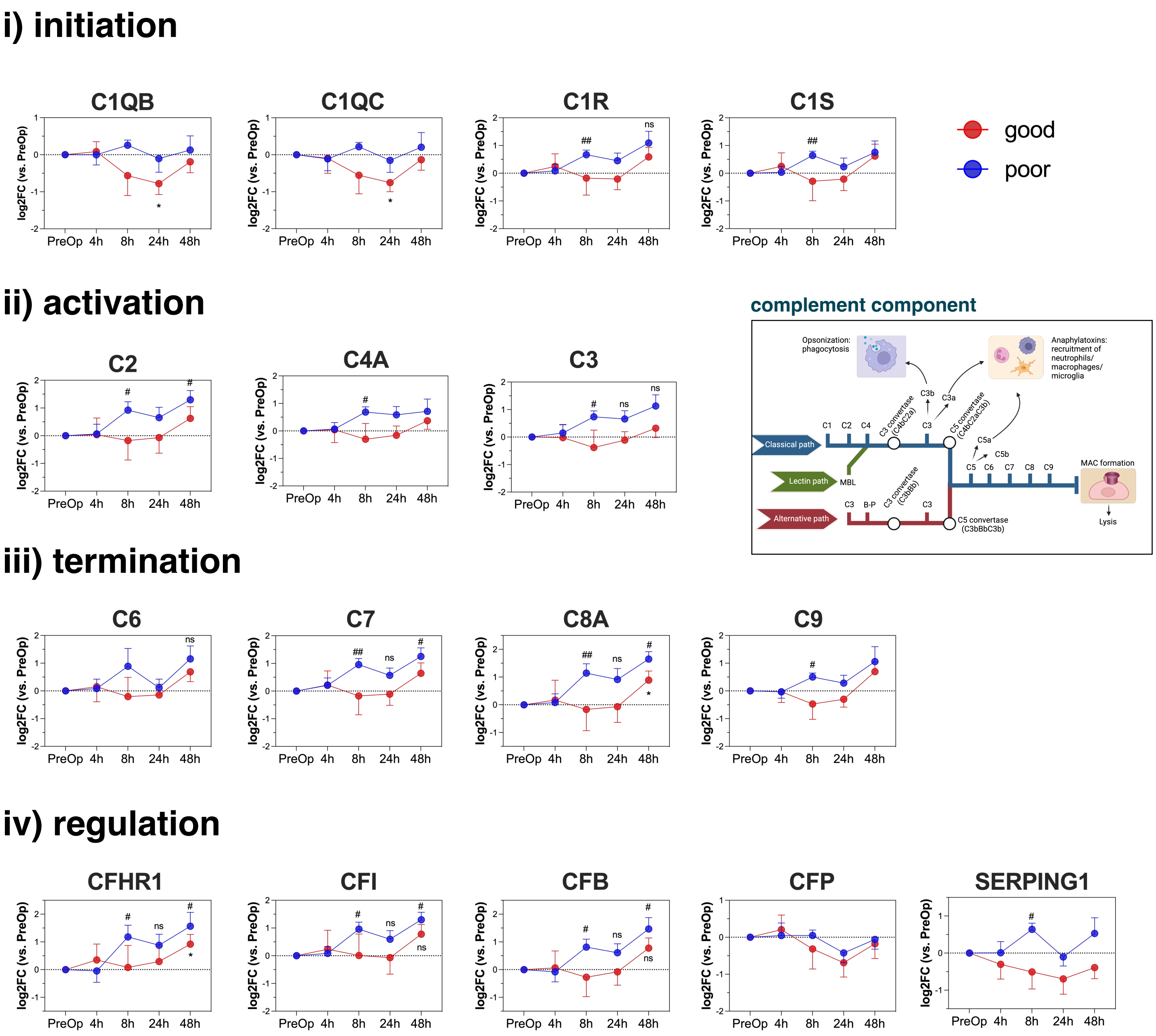

Supplementary Figure 5. Log2 normalized abundancies of the members of the complement system as determined in plasma-derived EVs by mass spectrometry relative to preoperative (PreOp) values (see Supplementary Methods). Statistical differences were tested by two-way ANOVA with repeated measures followed by multiple comparison analysis by Uncorrected Fisher's LSD: good *, $p<0.05$ (vs PreOp), poor #, $p < 0.05$, ## $p < 0.01$ (vs. PreOp), ns, $p < 0.1$. A schematic representation of the three main canonical complement pathways (classical, lectin and alternative) is included.
